# Exploring the Nonlinear Relationship Between Dietary Flavonoid Intake and Periodontitis

**DOI:** 10.1016/j.identj.2024.10.015

**Published:** 2024-11-14

**Authors:** Hua Li, Linlong Li, Shunbiao Yang, Wei Dai, Chunliang Guo, Guangyang Zhu, Zhi Wang, Zheng Wang, Xiaoqin Yan, Yun Liang

**Affiliations:** a Department of Pain, Jiangyou People's Hospital, Mianyang, China; b Department of Rehabilitation Medicine, Jiangyou People's Hospital, Mianyang, China; c College of Medicine, Southwest Jiaotong University, Chengdu, Sichuan, China; d Jiangyou People's Hospital, Mianyang, China

**Keywords:** Dietary flavonoid intake, Periodontitis, Oral health, NHANES

## Abstract

**Introduction and aims:**

Flavonoids are non-nutrient bioactive substances widely found in plants, possessing antioxidant and anti-inflammatory properties. Periodontitis is a long-term inflammatory disease that impacts the tissues supporting the teeth, poses a substantial burden on public health and individuals alike. This study aims to explore the association between dietary flavonoid intake and periodontitis.

**Methods:**

This study included 3005 participants from the 2009 to 2010 National Health and Nutrition Examination Survey. We compared the weighted prevalence of periodontitis across different participant groups. Binary logistic regression analysis was conducted to evaluate the relationship between dietary flavonoid intake and periodontitis. The restricted cubic spline plot was used to explore nonlinear relationships.

**Results:**

The prevalence of periodontitis among participants with total flavonoid intake in quartiles Q1 to Q4 was 54.95%, 44.11%, 40.62%, and 48.28%, respectively. When compared to the Q1 group of total flavonoid intake, the OR values for Q2 to Q4 groups were 0.58 (95% CI: 0.39-0.86, *P* = .01), 0.50 (95% CI: 0.35-0.73, *P* = .001), and 0.68 (95% CI: 0.50-0.91, *P* = .01), respectively. A significant nonlinear association was observed between ln-transformed total flavonoid intake and the likelihood of developing periodontitis (nonlinearity *P* < .001). The inflection point was identified at an ln-transformed total flavonoid intake of 4.05, corresponding to a total flavonoid intake of 57.54 mg. Beyond this inflection point, as the total flavonoid intake value continues to rise, there was a diminishing protective effect against periodontitis.

**Conclusions:**

Higher dietary flavonoid intake is associated with a reduced risk of periodontitis, with the greatest protective effect observed at moderate intake levels.

**Clinical Relevance:**

Understanding the association between flavonoid intake and periodontitis can guide dietary recommendations and interventions aimed at preventing periodontitis. This study supports the potential role of a flavonoid-rich diet in promoting periodontal health, suggesting that dietary modifications could be a viable strategy in periodontal disease prevention and management.

## Introduction

Periodontitis is a chronic inflammatory condition that affects the gums and supporting tissues surrounding the teeth. Its primary characteristics involve the development of periodontal pockets and the loss of alveolar bone, which can ultimately lead to tooth loss.^1^ Currently, the prevailing understanding of the pathogenic mechanism of periodontitis involves intricate interactions among active herpesviruses, dysregulated specific bacterial pathogens, and the host's immune response to inflammation.^2^ Periodontitis is likely to contribute to systemic diseases such as obesity, Alzheimer's disease, diabetes, inflammatory bowel disease, and cardiovascular diseases.^3^ Global statistics reveal that around 1.1 billion people suffer from severe periodontitis.^4^ This condition not only reduces the quality of life for affected individuals but also imposes a significant social and economic burden. Current periodontal therapy primarily consists of nonsurgical scaling, root planning, home care, and other supportive measures, while surgical treatments are typically reserved for advanced cases.^5^ Long-term maintenance is crucial for successful outcomes.

Given the considerable health risks associated with periodontitis and the intricacies involved in its treatment, early diagnosis, and prevention assume paramount significance. Adopting a healthy diet, reducing smoking, and maintaining good oral hygiene habits can serve as protective factors against preventing periodontal disease.^6^, ^7^, ^8^

Flavonoids are polyphenolic compounds found exclusively in plants, primarily classified into six subclasses: flavones, anthocyanins, flavonols, flavanones, flavanols, and isoflavones.^9^ They possess antioxidant and anti-inflammatory properties.^10^ Flavonoids are abundant in vegetables and fruits, particularly in foods like onions, tea, and soybeans.^11^ Dietary flavonoids have shown protective effects against various diseases including cardiovascular diseases, metabolic disorders, cancer, and diabetes.^10^^,^^12^^,^^13^ Moreover, numerous animal studies have highlighted their beneficial role in the treatment and prevention of periodontitis. For instance, recent study has shown that supplementation with citrus flavonoids significantly inhibits lipopolysaccharide-induced periodontitis in mice.^14^ Additionally, an animal experiment indicated that oral administration of flavonols compounds like quercetin could maintain periodontal homeostasis by modulating inflammatory responses and oral microbial composition.^15^ Certain clinical trials have reported positive impacts on clinical periodontal outcomes through dietary intervention as a nonsurgical adjunctive therapy in periodontal treatment, using substances such as tea, oolong tea, polyphenols, and flavonoids.^16^ Alhassani et al^17^ indicated that the consumption of flavonols might influence the risk of male periodontal disease. Liu et al^18^ reported an association between high total dietary flavonoid intake and decreased periodontal pocket depth as well as reduced clinical attachment loss. However, the connection between dietary flavonoid intake and periodontitis, especially concerning specific subclasses, has not been thoroughly studied.

Using data from the 2009 to 2010 National Health and Nutrition Examination Survey (NHANES), we performed a cross-sectional study to provide evidence regarding the link between dietary flavonoid intake and periodontal disease and to evaluate potential confounding factors influencing this relationship.

## Materials and methods

### Study design and participants

NHANES assesses the health and nutritional status of the U.S. noninstitutionalized population through various measures, including health interview surveys and laboratory tests. NHANES has a statistical design that employs stratified, multistage, probability sampling. All participants provided informed consent, and the study received approval from the National Center for Health Statistics Ethics Review Board.

NHANES collected information related to flavonoids compound intake through 24-hour dietary recall records during three cycles in 2007-2008, 2009-2010, and 2017-2018. In consideration of the introduction of the Full Mouth Periodontal Examination from 2009 to 2010 and continuing until 2013-2014, the only cycle in which flavonoids compound intake overlapped with periodontitis data were 2009-2010. Therefore, this study looked at the relevant data from 2009 to 2010 for analysis.

**Fig. 1:**
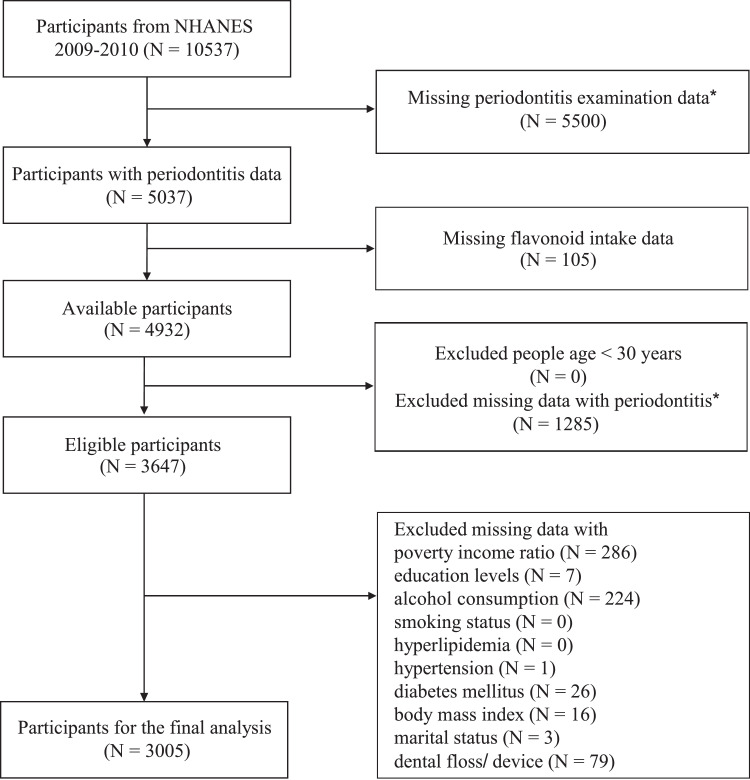
Flowchart of procedures for participants selection and inclusion. *Missing periodontitis examination data (*N* = 5500): The exclusion of individuals who did not undergo periodontal disease testing refers to participants who were initially not included in the assessment of their periodontal condition. Excluded missing data with periodontitis (*N* = 1285): The exclusion of individuals with missing periodontal disease data pertains to participants who agreed to undergo periodontal disease testing but, due to various reasons, did not participate in the actual testing, resulting in missing data for this specific variable.

During the 2009-2010 survey cycle, a total of 10,537 participants participated. The inclusion and exclusion criteria are provided in Figure 1. Ultimately, our analysis included 3005 eligible individuals. The study's findings represent a weighted population of 121,460,755 people.

### Assessment of flavonoids intake

Information on intake of total flavonoids and their subclasses was obtained from the U.S. Department of Agriculture's (USDA) Food and Nutritional Dietary Studies Database (FNDDS), which is linked to the NHANES database.^19^ The participants included in this study are the intersection between the two databases, and the data on flavonoid intake are collected from the same individuals who participated in NHANES. The FNDDS database estimates the levels of these flavonoids by coding all the collected foods, which are combined with the food consumption data from NHANES.^19^ The database provides intake values for 29 flavonoids, which can be categorized into six flavonoid subclasses (flavonols, flavones, flavan-3-ols, anthocyanidins, flavanones, and isoflavones). Flavonols encompass quercetin, myricetin, kaempferol, and isorhamnetin. Flavones consist of luteolin and apigenin. Flavan-3-ols encompass (−)-epicatechin 3-gallate, (−)-epicatechin, (−)-epigallocatechin 3-gallate, (−)-epigallocatechin, (+)-gallocatechin, (+)-catechin, theaflavin-3-gallate, theaflavin-3′-gallate, theaflavin-3,3′-digallate, theaflavin, and thearubigins. Anthocyanidins consist of delphinidin, cyanidin, malvidin, peonidin, pelargonidin, and petunidin. Flavanones include hesperetin, naringenin, and eriodictyol. Isoflavones comprise glycitein, genistein, and daidzein. Total flavonoid intake was defined as the sum of daily intake of various flavonoid compounds.

### Definition of periodontitis

In NHANES 2009-2010, periodontal exams were conducted only on adults aged 30 and above who had at least one natural tooth and had not recently taken antibiotics.^20^ Periodontitis was defined using the CDC and American Academy of Periodontology criteria, with probing depth and clinical attachment level as key indicators of periodontal examination. Participants in this study were classified into two groups: those with or without periodontitis, with the corresponding diagnostic criteria detailed in Appendix Table 1.^20^, ^21^, ^22^ Dental hygienists evaluate the periodontal condition of the participant in conjunction with the above diagnostic criteria.

### Assessment of covariates

Participants were grouped by age into 30 to 44, 45 to 54, 55 to 64, and 65+ years.^23^ Ethnicity was categorized as Hispanic, non-Hispanic black, non-Hispanic white, and other. Participants were divided into three categories according to their body mass index (BMI): those with a BMI under 25 kg/m² were classified as lean and normal weight, those with a BMI ranging from 25 to 30 kg/m² were considered overweight, and those with a BMI of 30 kg/m² or higher were categorized as obese. Participants were classified into three income groups based on the poverty income ratio (PIR): low (PIR < 1.3), middle (PIR 1.3-3.5), and high (PIR > 3.5). Education levels were grouped as less than high school, high school, and more than high school. Marital status was categorized as never married, widowed/divorced/separated, and married/living with a partner. Smoking status was assessed by determining whether a participant had smoked more than 100 cigarettes in their lifetime and was categorized as never, former, or current smoker.^24^ Drinking status was classified as never, former, light, moderate, or heavy drinking based on alcohol consumption of at least 12 times a year. The frequency of flossing and cleaning equipment use per week reflected participants’ oral hygiene practices, categorized as 0 to 1, 2 to 4, and 5 times or more.^25^ Diabetes mellitus (DM) was categorized as no, prediabetes, or diabetes. Hypertension and hyperlipidaemia were categorized as no or yes. Detailed diagnostic criteria for the three major chronic diseases can be found in Appendix Table 2.

### Statistical analyses

To produce nationally representative estimates, NHANES utilizes a complex, multistage, probability sampling survey design that incorporates dietary survey information. Therefore, we used the 24-hour dietary weight (Dietary Day One Sample Weight, WTDRD1) for the weighted analyses. In our study, categorical variables are described using frequencies and percentages for presentation. We used the *χ*² test to compare the characteristics of participants with and without periodontitis.

Due to the non-normal distribution of both total flavonoid intake and its six subclasses (anthocyanidins, flavonols, flavan-3-ols, flavones, flavanones, and isoflavones), data description was performed using quantiles. With the exception of isoflavones intake, where more than half the values were zero (*n* = 1716, 57.1%), thus categorized as ‘no or yes’, the remainder were grouped into quartiles, as detailed in Appendix Table 3. To explore the association of total flavonoid intake and its six subcategories with periodontitis, we constructed weighted multivariate logistic regression models. In the unadjusted regression model, we did not adjust for any covariates. Adjusted model 1 was adjusted for sex, age, and race. Adjusted model 2 included additional adjustments for BMI, PIR, drinking status, smoking status, marital status, education levels, weekly use of floss or cleaning equipment, DM, hypertension, and hyperlipidaemia.

**Fig. 2:**
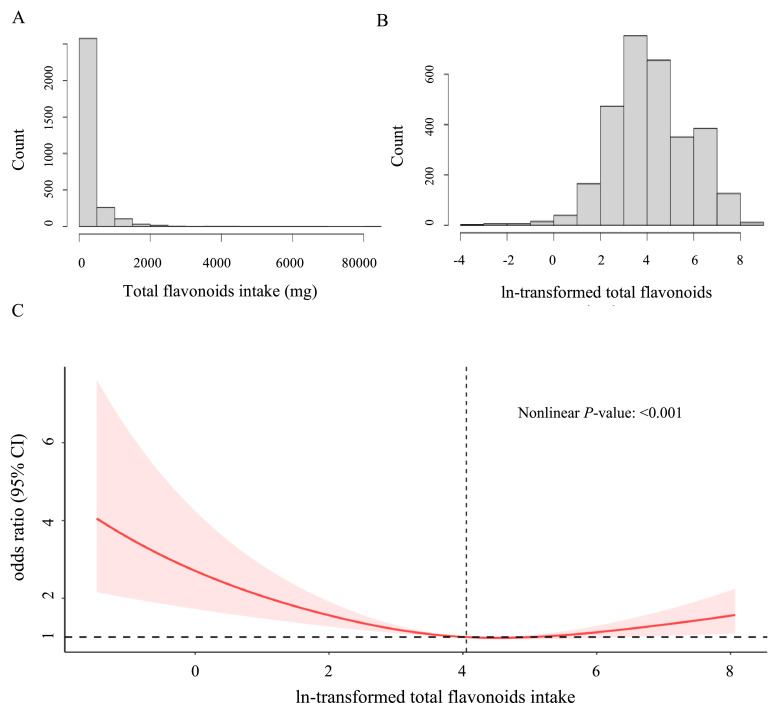
The distribution of total flavonoids intake (A). The distribution of ln-transformed total flavonoids intake (B). The full-adjusted relationship between ln-transformed total flavonoids intake and periodontitis using restricted cubic spline (C). The solid line represents the fitted nonlinear curve. The area adjacent to the solid line represents the 95% confidence interval. CI, confidence interval.

Then, the nonlinear association between total flavonoid intake and periodontitis was evaluated by a restricted cubic spline method. It's worth noting that in logistic regression when a continuous variable is used as the independent variable, it's required to establish a linear relationship between that variable and logit(p). However, our analysis indicated that the linear relationship was not present between total flavonoid intake (represented as a continuous variable) and logit(p). As a result, we performed an ln-transformation on total flavonoid intake. The histograms before and after transformation are illustrated in Figure 2A,B, respectively. The restricted cubic spline analysis incorporated three strategically positioned knots along the curve. Subsequent analyses examined the link between total flavonoid intake and periodontitis across different subgroups. This approach enabled separate observations of associations with periodontitis in each subgroup, assessing their consistency. During the data screening process, a significant proportion of data was found to be missing for alcohol consumption (*N* = 224) and PIR (*N* = 286). To address the potential source of selection bias arising from this missing data, a sensitivity analysis was conducted, which involved excluding data related to PIR and alcohol consumption. Consequently, the sensitivity analysis encompassed a total of 3515 participants. Furthermore, collinearity diagnostics were carried out to assess the presence of multicollinearity. There is no severe collinearity, with variance inflation factor values consistently below 2. A variance inflation factor exceeding 10 would be indicative of severe collinearity.^26^ Statistical significance was set at *P* < .05. All analyses were performed using nhanesR (version 0.9.4.3) and R studio (version 4.3.1). This study was conducted following the STROBE Guidelines.

## Results

### Descriptive characteristics

Based on our inclusion and exclusion criteria, our study ultimately included 3005 eligible individuals (Figure 1). Table 1 provides the descriptive characteristics of the population, categorized by periodontitis status. The prevalence of periodontitis was analysed with weighted data. The periodontitis prevalence rates among participants in the total flavonoid intake Q1 to Q4 groups were 54.95%, 44.11%, 40.62%, and 48.28%, respectively. Notably, a significant difference in periodontitis prevalence was observed among these groups (*P* = .005). The intake of flavonols, flavan-3-ols, and flavones in the Q1 to Q4 groups showed no significant differences in periodontitis prevalence (*P* > .05), suggesting no association between these intakes and the occurrence of periodontitis. However, among participants in the anthocyanidins and flavanones intake Q1 groups, as well as those who did not consume isoflavones, a significantly higher prevalence of periodontitis was observed (*P* < .05), suggesting an association between these intakes and the occurrence of periodontitis. As participants’ age increased, there was a progressive rise in the prevalence of periodontitis, starting at 30.59% among those aged 30 to 44 years and reaching 68.43% among participants aged 65 years and older (*P* < .001). Notably, females exhibited a significantly lower prevalence of periodontitis compared to males (37.38% vs 55.95%, *P* < .001). Furthermore, specific factors were linked to a lower prevalence of periodontitis, including non-Hispanic white ethnicity, being married or living with a partner, having a PIR greater than 3.5, possessing an education level above high school, never smoking, not having hypertension, not having DM, or flossing and using cleaning equipment 2 to 4 days a week.

**Table 1:** Descriptive characteristics of the study population stratified by periodontitis.

Characteristic	Total	No periodontitis	Periodontitis	*P* value*
	(*N* = 3005)	(*N* = 1329)	(*N* = 1676)	
**Total flavonoid intake, *n* (%)**				.005
Q1	751 (24.99)	299 (45.05)	452 (54.95)	
Q2	752 (25.03)	340 (55.89)	412 (44.11)	
Q3	751 (24.99)	355 (59.38)	396 (40.62)	
Q4	751 (24.99)	335 (51.72)	416 (48.28)	
**Flavonols intake, *n* (%)**				.578
Q1	753 (25.06)	315 (51.44)	438 (48.56)	
Q2	750 (24.96)	330 (51.01)	420 (48.99)	
Q3	751 (24.99)	353 (56.17)	398 (43.83)	
Q4	751 (24.99)	331 (53.86)	420 (46.14)	
**Flavones intake, *n* (%)**				.446
Q1	777 (25.86)	314 (49.75)	463 (50.25)	
Q2	728 (24.23)	324 (53.26)	404 (46.74)	
Q3	751 (24.99)	332 (53.77)	419 (46.23)	
Q4	749 (24.92)	359 (55.73)	390 (44.27)	
**Flavan-3-ols intake, *n* (%)**				.053
Q1	750 (24.96)	304 (46.63)	446 (53.37)	
Q2	752 (25.02)	331 (54.98)	421 (45.02)	
Q3	751 (24.99)	355 (57.25)	396 (42.75)	
Q4	752 (25.03)	339 (53.17)	413 (46.83)	
**Anthocyanidins intake, *n* (%)**				<.001
Q1	969 (32.25)	374 (43.89)	595 (56.11)	
Q2	534 (17.77)	228 (54.76)	306 (45.24)	
Q3	751 (24.99)	352 (56.42)	399 (43.58)	
Q4	751 (24.99)	375 (59.24)	376 (40.76)	
**Flavanones intake, *n* (%)**				.015
Q1	1006 (33.48)	406 (48.73)	600 (51.27)	
Q2	495 (16.47)	226 (55.76)	269 (44.24)	
Q3	754 (25.09)	377 (60.07)	377 (39.93)	
Q4	750 (24.96)	320 (49.60)	430 (50.40)	
**Isoflavones intake, *n* (%)**				.012
No	1716 (57.11)	710 (49.96)	1006 (50.04)	
Yes	1289 (42.89)	619 (57.28)	670 (42.72)	
**Age, *n* (%)**				<.001
30-44	1046 (34.81)	651 (69.41)	395 (30.59)	
45-54	704 (23.43)	308 (53.47)	396 (46.53)	
55-64	587 (19.53)	192 (40.63)	395 (59.37)	
≥65	668 (22.23)	178 (31.57)	490 (68.43)	
**Sex, *n* (%)**				<.001
Female	1446 (48.12)	791 (62.62)	655 (37.38)	
Male	1559 (51.88)	538 (44.05)	1021 (55.95)	
**Race/Ethnicity, *n* (%)**				<.001
Non‐Hispanic White	1543 (51.35)	782 (57.63)	761 (42.37)	
Non‐Hispanic Black	534 (17.77)	189 (37.80)	345 (62.20)	
Hispanic	798 (26.56)	306 (41.93)	492 (58.07)	
Other Race	130 (4.33)	52 (45.67)	78 (54.33)	
**BMI, *n* (%)**				.053
Underweight/normal	747 (24.86)	345 (57.70)	402 (42.30)	
Overweight	1042 (34.68)	457 (52.00)	585 (48.00)	
Obese	1216 (40.46)	527 (51.10)	689 (48.90)	
**Marital status, *n* (%)**				.02
Married/living with partner	1988 (66.16)	908 (55.24)	1080 (44.76)	
Never married	290 (9.65)	141 (50.84)	149 (49.16)	
Widowed/divorced/separated	727 (24.19)	280 (47.24)	447 (52.76)	
**PIR, *n* (%)**				<.001
<1.3	827 (27.52)	269 (35.24)	558 (64.76)	
1.3-3.5	1139 (37.90)	467 (46.95)	672 (53.05)	
>3.5	1039 (34.58)	593 (63.46)	446 (36.54)	
**Education levels, *n* (%)**				<.001
Below high school	303 (10.08)	77 (28.34)	226 (71.66)	
High school	1132 (37.67)	399 (43.35)	733 (56.65)	
Above high school	1570 (52.25)	853 (60.27)	717 (39.73)	
**Alcohol consumption, *n* (%)**				.089
Never	334 (11.12)	136 (48.11)	198 (51.89)	
Former	535 (17.80)	188 (47.10)	347 (52.90)	
Mild	1076 (35.81)	489 (54.06)	587 (45.94)	
Moderate	461 (15.34)	265 (60.12)	196 (39.88)	
Heavy	599 (19.93)	251 (52.37)	348 (47.63)	
**Smoking status, *n* (%)**				<.001
Never	1612 (53.64)	837 (61.79)	775 (38.21)	
Former	806 (26.82)	313 (46.80)	493 (53.20)	
Current	587 (19.53)	179 (36.03)	408 (63.97)	
**Hyperlipidaemia, *n* (%)**				.263
No	713 (23.73)	350 (55.52)	363 (44.48)	
Yes	2292 (76.27)	979 (52.44)	1313 (47.56)	
**Hypertension, *n* (%)**				<.001
No	1692 (56.31)	863 (59.35)	829 (40.65)	
Yes	1313 (43.69)	466 (43.24)	847 (56.76)	
**Diabetes mellitus, *n* (%)**				<.001
No	2208 (73.48)	1071 (56.64)	1137 (43.36)	
Prediabetes	243 (8.09)	100 (47.67)	143 (52.33)	
DM	554 (18.43)	158 (36.29)	396 (63.71)	
**Flossing, *n* (%)**				<.001
0-1 d a week	1220 (40.60)	431 (44.31)	789 (55.69)	
2-4 d a week	717 (23.86)	373 (60.80)	344 (39.20)	
≥5 d a week	1068 (35.54)	525 (56.75)	543 (43.25)	

BMI, body mass index; PIR, poverty income ratio.

⁎ *P*-value by chi-square test for classified variables.

### Associations between flavonoid intake and periodontitis

Binary logistic regression models were employed to explore the association between total flavonoid intake and its six subclasses and the prevalence of periodontitis, as presented in Table 2. Regarding total flavonoid intake, when compared to the Q1 group, participants in the Q2 to Q4 groups exhibited an OR value consistently less than 1 in all three regression models (*P* < .05). This indicates that a certain level of flavonoid intake in the diet was associated with a reduced likelihood of periodontitis. After adjusting for all covariates, when compared to the Q1 group of total flavonoid intake, the OR values for Q2 to Q4 groups were 0.58 (95% CI: 0.39-0.86, *P* = .01), 0.50 (95% CI: 0.35-0.73, *P* = .001), and 0.68 (95% CI: 0.50-0.91, *P* = .01), respectively.

**Table 2:** Adjusted association of flavonoids intake with periodontitis.

	Odds ratio (95% CI) associated with periodontitis		
Exposure	Unadjusted model*	Adjust 1†	Adjust 2‡
**Total flavonoid intake**			
Q1	1 (Ref)	1 (Ref)	1 (Ref)
Q2	0.65 (0.49,0.86); 0.01	0.53 (0.35, 0.80); 0.01	0.58 (0.39, 0.86); 0.01
Q3	0.56 (0.40,0.78); 0.002	0.42 (0.28, 0.64); 0.002	0.50 (0.35, 0.73); 0.001
Q4	0.77 (0.59,1.00); 0.05	0.57 (0.40, 0.81); 0.01	0.68 (0.50, 0.91); 0.01
**Flavonols intake**			
Q1	1 (Ref)	1 (Ref)	1 (Ref)
Q2	1.02 (0.71,1.45); 0.92	0.83 (0.56, 1.23); 0.29	0.92 (0.63, 1.35); 0.65
Q3	0.83 (0.52,1.31); 0.39	0.69 (0.40, 1.19); 0.15	0.80 (0.50, 1.27); 0.32
Q4	0.91 (0.66,1.24); 0.52	0.71 (0.51, 1.00); 0.05	0.79 (0.57, 1.09); 0.13
**Flavones intake**			
Q1	1 (Ref)	1 (Ref)	1 (Ref)
Q2	0.87 (0.63,1.20); 0.36	0.82 (0.54, 1.24); 0.28	0.94 (0.66, 1.34); 0.7
Q3	0.85 (0.57,1.27); 0.4	0.75 (0.46, 1.22); 0.2	0.87 (0.55, 1.37); 0.52
Q4	0.79 (0.61,1.02); 0.06	0.68 (0.53, 0.86); 0.01	0.86 (0.70, 1.05); 0.14
**Flavan-3-ols intake**			
Q1	1 (Ref)	1 (Ref)	1 (Ref)
Q2	0.72 (0.58,0.89); 0.01	0.66 (0.50, 0.87); 0.01	0.73 (0.55, 0.97); 0.03
Q3	0.65 (0.53,0.80); <0.001	0.52 (0.39, 0.70); 0.002	0.65 (0.48, 0.87); 0.01
Q4	0.77 (0.58,1.02); 0.07	0.60 (0.45, 0.81); 0.01	0.70 (0.54, 0.91); 0.01
**Anthocyanidins intake**			
Q1	1 (Ref)	1 (Ref)	1 (Ref)
Q2	0.65 (0.46,0.91); 0.02	0.59 (0.40, 0.87); 0.02	0.74 (0.54, 1.01); 0.05
Q3	0.60 (0.52,0.70); <0.001	0.54 (0.42, 0.69); <0.001	0.64 (0.50, 0.81); <0.001
Q4	0.54 (0.44,0.66); <0.001	0.48 (0.36, 0.64); <0.001	0.65 (0.51, 0.82); 0.001
**Flavanones intake**			
Q1	1 (Ref)	1 (Ref)	1 (Ref)
Q2	0.75 (0.49,1.17); 0.19	0.74 (0.45, 1.23); 0.2	0.78 (0.48, 1.29); 0.31
Q3	0.63 (0.47,0.85); 0.005	0.61 (0.40, 0.91); 0.02	0.76 (0.51, 1.13); 0.16
Q4	0.97 (0.69,1.36); 0.83	0.77 (0.52, 1.16); 0.17	0.95 (0.62, 1.47); 0.82
**Isoflavones intake**			
No	1 (Ref)	1 (Ref)	1 (Ref)
Yes	0.74 (0.60,0.93); 0.01	0.72 (0.55, 0.95); 0.03	0.80 (0.63, 1.01); 0.06

CI, confidence interval.

⁎ Unadjusted model: nonadjusted model.

† Adjust 1: Adjust for age, sex, race.

‡ Adjust 2: Adjust for age, sex, race, body mass index, poverty income ratio, education levels, marital status, smoking status, alcohol consumption, hyperlipidaemia, hypertension, diabetes mellitus, and flossing.

In the adjusted Model 2, the OR values for flavonols, flavones, and flavanones intake in the Q2 to Q4 groups were not statistically significant (*P* > .05). For flavan-3-ols intake, the OR values in the Q2 to Q4 groups were 0.73 (95% CI: 0.55-0.97, *P* = .03), 0.65 (95% CI: 0.48-0.87, *P* = .01), and 0.70 (95% CI: 0.54-0.91, *P* = .01), respectively. In the case of Anthocyanidins intake, the OR values in the Q2 to Q4 groups were 0.74 (95% CI: 0.54-1.01, *P* = .05), 0.64 (95% CI: 0.50-0.81, *P* < .001), and 0.65 (95% CI: 0.51-0.82, *P* = .001), respectively. When compared to participants who did not consume isoflavones, the OR values for periodontitis associated with isoflavones intake were 0.72 (95% CI: 0.55, 0.95, *P* = .03) in adjusted model 1 and 0.80 (95% CI: 0.63, 1.01, *P* = .06) in adjusted model 2.

To investigate whether the impact of total flavonoid intake on periodontitis is potentially attributed to dietary health habits, we computed healthy eating index (HEI) scores using the same criteria as described in their study.^27^ When additionally adjusting for the HEI, the OR values associated with periodontitis for total flavonoid intake Q2 to Q4 groups were 0.58 (95% CI: 0.38-0.88, *P* = .01), 0.50 (95% CI: 0.32-0.77, *P* = .004), and 0.67 (95% CI: 0.49-0.93, *P* = .02). If HEI was not adjusted, the ORs were 0.58 (95% CI: 0.39-0.86, *P* = .01), 0.50 (95% CI: 0.35-0.73, *P* = .001), and 0.68 (95% CI: 0.50-0.91, *P* = .01). These results suggest that the influence of HEI on the relationship between flavonoid intake and periodontitis is relatively minor.

### Nonlinear relationships

A significant nonlinear association was observed between ln-transformed total flavonoid intake and the likelihood of developing periodontitis (nonlinearity *P* < .001), as illustrated in Figure 2C. The inflection point was identified at an ln-transformed total flavonoid intake of 4.05, corresponding to a total flavonoid intake of 57.54 mg. This value represents the point of lowest likelihood for periodontitis. Notably, this total flavonoid intake of 57.54 aligns with the Q3 group (56.58-202.57), which exhibited the lowest periodontitis prevalence as indicated in Table 1. Beyond this inflection point, as the total flavonoid intake value continues to rise, there was a diminishing protective effect against periodontitis.

### Subgroup analyses and sensitivity analysis

After controlling for all covariates, the subgroup analysis revealed no significant effect modification (*P* value for interaction >0.05) within the sex, age, BMI, smoking status, alcohol consumption, hyperlipidaemia, and DM subgroups (Table 3). This suggests that the impact of total flavonoid intake on periodontitis does not significantly differ across these subgroups. However, a significant interaction was observed in the hypertension subgroups, with a *P* value of 0.021. In other words, the effect of total flavonoid intake on periodontitis significantly differs between participants with and without hypertension. Total flavonoid intake can reduce the likelihood of periodontitis exclusively in participants without hypertension. Sensitivity analysis, as presented in Appendix Table 4, was conducted to validate the robustness of our results. The findings from the sensitivity analysis were consistent with those presented in Table 2. Participants were further divided into groups of moderate/severe periodontitis and no/mild periodontitis. The logistic regression results are shown in Appendix Table 5. Consistent with the previous findings, increased flavonoid intake was linked to a lower likelihood of moderate/severe periodontitis.

**Table 3:** Subgroup analysis of adjusted association of flavonoids intake with periodontitis.

	Adjusted odds ratio (95% CI); P*			
Subgroups	Q2	Q3	Q4	Sgnificanc (P) for interaction
**Sex**				.216
Female	0.76 (0.50, 1.17); .193	0.64 (0.37, 1.09); .094	0.80 (0.56, 1.14); .197	
Male	0.43 (0.25, 0.74); .004	0.39 (0.24, 0.64); <.001	0.56 (0.34, 0.91); .022	
**Age**				.081
30-44	0.67 (0.41, 0.10); .108	0.50 (0.30, 0.82); .009	0.63 (0.33, 1.20); .146	
45-54	0.38 (0.14, 1.04); .057	0.29 (0.13, 0.63); .004	0.41 (0.21, 0.82); .015	
55-64	0.54 (0.22, 1.30); .156	0.59 (0.27, 1.30); .173	1.23 (0.49, 3.10); .648	
≥65	1.04 (0.60, 1.79); .889	1.25 (0.70, 2.23); .426	0.99 (0.63, 1.55); .945	
**Body mass index**				.565
Underweight/normal	0.86 (0.36, 2.03); .713	0.77 (0.30, 1.96); .563	0.97 (0.44, 2.15); .944	
Overweight	0.61 (0.35, 1.07); .080	0.44 (0.26, 0.76); .005	0.55 (0.32, 0.95); .034	
Obese	0.43 (0.25, 0.72); .003	0.44 (0.30, 0.63); <.001	0.65 (0.43, 1.00); .048	
**Smoking status**				.369
Never	0.53 (0.33, 0.87); .014	0.48 (0.31, 0.76); .004	0.62 (0.39, 0.98); .042	
Former	0.56 (0.29, 1.07); .076	0.36 (0.20, 0.62); .001	0.74 (0.36, 1.53); .391	
Current	0.75 (0.36, 1.57); .424	1.21 (0.39, 3.78); .73	0.61 (0.25, 1.51); .267	
**Alcohol consumption**				.208
Never	0.43 (0.17, 1.06); .064	0.56 (0.28, 1.11); .092	0.48 (0.26, 0.89); .023	
Former	0.43 (0.18, 1.04); .060	0.39 (0.14, 1.07); .066	0.45 (0.21, 0.96); .040	
Mild	0.44 (0.26, 0.76); .006	0.49 (0.27, 0.91); .027	0.86 (0.46, 1.60); .616	
Moderate	1.31 (0.57, 3.04); .504	0.73 (0.24, 2.21); .550	0.65 (0.28, 1.50); .286	
Heavy	0.75 (0.29, 1.96); .534	0.56 (0.34, 0.92); .024	0.59 (0.32, 1.09); .086	
**Hyperlipidaemia**				.823
No	0.40 (0.15, 1.10); .074	0.37 (0.20, 0.67); .003	0.48 (0.22, 1.04); .062	
Yes	0.64 (0.45, 0.92); .017	0.57 (0.39, 0.82); .005	0.73 (0.57, 0.93); .013	
**Hypertension**				**.021**
No	0.46 (0.30, 0.73); .002	0.35 (0.22, 0.55); <.001	0.44 (0.29, 0.69); .001	
Yes	0.85 (0.51, 1.43); .518	0.87 (0.52, 1.45); .573	1.26 (0.88, 1.82); .191	
**Diabetes mellitus**				.101
No	0.52 (0.32, 0.85); .012	0.43 (0.29, 0.64); <.001	0.55 (0.34, 0.89); .018	
Prediabetes	0.95 (0.29, 3.15); .925	0.85 (0.24, 2.92); .777	3.54 (1.49, 8.41); .007	
DM	1.02 (0.44, 2.34); .971	1.01 (0.46, 2.23); .970	1.17 (0.64, 2.12); .589	

CI, confidence interval.

p < 0.05 is indicated in bold.

⁎ We used the lowest quartile as the reference category. Adjust for age, sex, race, body mass index, poverty income ratio, education levels, marital status, smoking status, alcohol consumption, hyperlipidaemia, hypertension, diabetes mellitus, and flossing, but not for the specific stratification variables of interest.

## Discussion

Our research findings suggest that individuals with a higher total flavonoid intake (Q2-Q4 vs Q1 group) exhibit a lower prevalence of periodontitis, and higher total flavonoid intake is associated with a decreased likelihood of periodontitis. Furthermore, there is a significant nonlinear relationship between total flavonoid intake and periodontitis, with the lowest theoretical prevalence of periodontitis occurring at a total flavonoid intake of 57.54 mg. Among the six subclasses of total flavonoids, greater consumption of flavan-3-ols and anthocyanidins is also significantly linked to a reduced likelihood of periodontitis.

Our analysis shows significant differences in periodontitis prevalence across various socio-demographic groups. We observed a lower periodontitis prevalence among younger individuals, females, non-Hispanic white participants, those with better socioeconomic status, and higher educational attainment. These findings are in line with earlier investigations using NHANES data,^27^, ^28^, ^29^ highlighting the need to consider covariates when assessing the link between total flavonoid intake and periodontitis. Eke et al used the NHANES database to investigate periodontitis prevalence among U.S. adults, finding that more than 47% of the sample, equivalent to 64.7 million individuals, were affected by periodontitis. The distribution was 8.7% with mild, 30.0% with moderate, and 8.5% with severe periodontitis. In our study, 46.8% of participants were found to have periodontitis, representing 64.6 million adults. Among them, the proportions of mild, moderate, and severe periodontitis were 9.2%, 29.5%, and 8.1%, respectively. This suggests that our statistical analysis is reliable. The minor discrepancies may be attributed to differences in study participants and inclusion/exclusion criteria.^20^ Additionally, our results indicate a higher prevalence of periodontitis among participants with hypertension or DM, suggesting that these conditions can affect periodontal health. Muñoz Aguilera et al^30^ conducted a systematic review and meta-analysis to investigate the association between hypertension and periodontitis, and their findings corroborate our study. Furthermore, a review study has indicated that DM represents a potential risk factor for periodontitis.^31^

In recent years, there has been significant scholarly attention on the relationship between periodontitis and dietary habits. Li et al^27^ employed the HEI as a measure of dietary health and discovered that participants with higher HEI scores exhibited a reduced risk of periodontitis. Similarly, Machado et al^32^ utilized the dietary inflammatory index to assess the inflammatory potential of diets and found evidence of an association between an inflammatory diet and periodontitis. Furthermore, insufficient or excessive intake of vitamin C can both elevate the risk of periodontitis.^33^ In our previous investigations, we observed a connection between the consumption of probiotics and a decreased likelihood of periodontitis.^6^ These results demonstrate that dietary factors play an important role in periodontitis risk and prevention.

Flavonoids are commonly found in plants and plant-based beverages, such as various fruits, vegetables, as well as coffee, tea, and red wine, among others. For instance, berries, especially blueberries, are rich in anthocyanidins, while green tea contains high levels of flavan-3-ols, such as catechins. Citrus fruits are known for their flavanone content. For more detailed information on the flavonoid content of specific foods, the USDA's ‘Flavonoid Database’ can be consulted, which provides values for different food items and facilitates the estimation of flavonoid intakes. The web address for this database is provided in the Data Availability Statement.

Several previous studies have indicated that dietary consumption of specific fruits, vegetables, coffee, and tea, may reduce the risk of periodontal disease.^34^, ^35^, ^36^, ^37^ Currently, numerous cell experiments and animal models have also demonstrated that flavonoids can inhibit the expression of various inflammatory factors, exerting anti-inflammatory effects and potentially reducing the risk of several inflammatory diseases, including periodontitis.^14^^,^^15^^,^^38^ Specifically, citrus flavonoids such as eriocitrin and eriodictyol have been shown to significantly inhibit gingival inflammation by reducing the levels of proinflammatory cytokines IL-1β and TNF-α, while increasing the anti-inflammatory cytokine IL-10.^14^ This modulation aids in reducing inflammatory cell infiltration and preserving connective tissue. Additionally, flavonoids are potent antioxidants, which can enhance the activities of enzymes like SOD, CAT, and GPx, thereby mitigating oxidative stress and damage.^14^ Recent research has shown that flavonoids are capable of inhibiting key regulatory enzymes or transcription factors that play crucial roles in managing mediators of inflammation.^38^ Furthermore, quercetin, a common flavonoid, has been demonstrated to foster a balanced oral microbiota by reducing the abundance of pathogenic species and promoting nonpathogenic bacteria, contributing to periodontal health.^15^ These findings collectively highlight the multifaceted role of flavonoids in modulating inflammation, oxidative stress, and microbial composition, providing a robust biological basis for their protective effects against periodontal disease. Furthermore, Tanaka et al^39^ identified a reduced risk of periodontal disease in young females associated with the consumption of soy and isoflavones. The study by Alhassani et al^17^ revealed that the intake of a certain amount of flavonols may reduce the risk of self-reported periodontal disease in men. However, previous studies had some limitations, such as their focus solely on a specific subclass of flavonoids or the intake of specific plants or plant-based beverages. Additionally, some were conducted within specific populations or relied on self-reported methods to diagnose periodontitis. In contrast, this study is a large-scale cross-sectional investigation based on a representative weighted population, examining the relationship between flavonoid and its six subclasses and periodontitis assessed by dental professionals. Our study addresses these limitations, thus offering a more comprehensive analysis, which is one of the strengths of our research. Additionally, to the best of our knowledge, this is the first study to explore the nonlinear relationship between total flavonoid intake and periodontitis.

This study benefits from a substantial sample size, including 3005 participants, enhancing the robustness of the findings. The use of weighted data from the NHANES conducted between 2009 and 2010 ensures the study's relevance to the U.S. population. The study pioneers in exploring the nonlinear relationship between total flavonoid intake and periodontitis, contributing novel insights to the field. This study has a few limitations. First, its cross-sectional design does not allow for establishing causality between total flavonoid intake and periodontitis prevalence. Secondly, the findings mainly apply to the U.S. population. Thirdly, our methodology did not include the intake of proanthocyanidins, which may have impacted the comprehensiveness of our analysis. Proanthocyanidins are a type of flavonoid known for their anti-inflammatory and antioxidant properties, which may contribute to periodontal health. The exclusion of proanthocyanidins intake may have led to an underestimation of the overall impact of flavonoid intake on periodontitis, as their beneficial effects were not accounted for in our findings. Additionally, due to the inclusion and exclusion criteria applied, the final sample may not be fully representative of the entire U.S. population but rather represents the relevant subpopulation based on the applied weights.

## Conclusions

In conclusion, our study suggests that higher dietary flavonoid intake (Q2-Q4 vs Q1) is associated with a reduced likelihood of periodontitis, highlighting the potential benefits of a flavonoid-rich diet in promoting oral health. Furthermore, we uncover a nonlinear relationship between dietary flavonoid intake and periodontitis. However, further research is needed to establish causal relationships and generalizability beyond the U.S. population.

## Conflict of interest

None of the authors have any potential conflicts of interest.

## Appendix Supplementary materials

Image, application 1

Image, application 2

Image, application 3

## Footnotes

Supplementary material associated with this article can be found in the online version at doi:10.1016/j.identj.2024.10.015.
