# Supplementary material for: Exploring the Nonlinear Relationship Between Dietary Flavonoid Intake and Periodontitis
Source: Int Dent J. 2024 Nov 14;75(2):716–26. doi: 10.1016/j.identj.2024.10.015 (PMC11976537; doi:10.1016/j.identj.2024.10.015)
Supplement: Supplementary file 1 [file mmc1.docx]

**Appendix Table 1**. Periodontitis classification criteria according to the probing depth (PD) and attachment loss (AL).

| **Classification** | **Evaluation Criteria** |
| --- | --- |
| No periodontitis | No evidence of mild, moderate, or severe periodontitis |
| Mild periodontitis | ≥2 interproximal sites with AL ≥3 mm, and ≥2 interproximal sites with PD ≥4 mm (not on the same tooth) or one site with PD ≥5 mm |
| Moderate periodontitis | ≥2 interproximal sites with AL ≥4 mm (not on the same tooth), or ≥2 interproximal sites with PD ≥5 mm (not on the same tooth) |
| Severe periodontitis | ≥2 interproximal sites with AL ≥6 mm (not on the same tooth) and ≥1 interproximal site with PD ≥5 mm |

**Appendix Table 2**. The detailed overview of how we obtained information on hypertension, hyperlipidemia, and diabetes.

| **Hypertension** | The inclusion of hypertension was determined by considering both the results of a questionnaire survey and three separate blood pressure measurements taken from the participants. The participants were asked the following questions: " {Have you/Has SP} ever been told by a doctor or other health professional that {you/s/he} had hypertension, also called high blood pressure? Because of {your/SP's} (high blood pressure/hypertension), {have you/has s/he} ever been told to . . . take prescribed medicine?” And Hypertension was also confirmed if the blood pressure measurements exceeded 140/90 mmHg. |
| --- | --- |
| **Hyperlipidemia** | The information regarding hyperlipidemia is primarily obtained based on the following criteria  1. High Triglycerides (TG): TG levels greater than or equal to 150 mg/dL.  2. High Total Cholesterol (TC): TC levels greater than or equal to 200 mg/dL [5.18 mmol/L].  3. Elevated Low-density lipoprotein (LDL)-Cholesterol: LDL cholesterol levels greater than or equal to 130 mg/dL [3.37 mmol/L].  4. Low high-density lipoprotein (HDL)-Cholesterol: In males, HDL cholesterol levels below 40 mg/dL [1.04 mmol/L]. In females, HDL cholesterol levels below 50 mg/dL [1.30 mmol/L].  5. The use of lipid-lowering medications. |
| **Diabetes mellitus (DM)** | The diagnostic criteria for diabetes are:  1. doctor told you have diabetes ({Other than during pregnancy, {have you/has SP}/ {Have you/Has SP}} ever been told by a doctor or health professional that {you have/{he/she/SP} has} diabetes or sugar diabetes?),  2. glycohemoglobin HbA1c (%) >= 6.5,  3. fasting glucose (mmol/l) >= 7.0,  4. random blood glucose (mmol/l) >= 11.1,  5. two-hour OGTT blood glucose (mmol/l) >= 11.1,  6. Use of diabetes medication or insulin,  Prediabetes: impaired fasting glycaemia and impaired glucose tolerance. |

**Appendix Table 3**. Details of flavonoids intake division.

| **Variables** | **Division details** | **Corresponding range (mg)** | **Frequency** | **Percentage** |
| --- | --- | --- | --- | --- |
| **Total flavonoid intake** | Q1 | [0, 20.81] | 751 | 24.99% |
|  | Q2 | (20.81, 56.58] | 752 | 25.03% |
|  | Q3 | (56.58, 202.57] | 751 | 24.99% |
|  | Q4 | (202.57, 8018.67] | 751 | 24.99% |
| **Isoflavones intake** | NO (=0) | [0, 0] | 1716 | 57.1% |
|  | Yes (>0) | (0, 216.75] | 1289 | 42.9% |
| **Anthocyanidins intake** | Q1 | [0, 0] | 969 | 32.25% |
|  | Q2 | (0, 1.19] | 534 | 17.77% |
|  | Q3 | (1.19, 9.06] | 751 | 24.99% |
|  | Q4 | (9.06, 454.42] | 751 | 24.99% |
| **Flavan-3-ols intake** | Q1 | [0, 3.69] | 750 | 24.96% |
|  | Q2 | (3.69, 12.89] | 752 | 25.02% |
|  | Q3 | (12.89, 62.94] | 751 | 24.99% |
|  | Q4 | (62.94, 7727.37] | 752 | 25.02% |
| **Flavanones intake** | Q1 | [0, 0] | 1006 | 33.48% |
|  | Q2 | (0, 0.35] | 495 | 16.47% |
|  | Q3 | (0.35, 9.88] | 754 | 25.09% |
|  | Q4 | (9.88, 522.72] | 750 | 24.96% |
| **Flavones**  **intake** | Q1 | [0, 0.13] | 777 | 25.86% |
|  | Q2 | (0.13, 0.46] | 728 | 24.23% |
|  | Q3 | (0.46, 1.1] | 751 | 24.99% |
|  | Q4 | (1.1, 87.93] | 749 | 24.93% |
| **Flavonols intake** | Q1 | [0, 6.41] | 753 | 25.06% |
|  | Q2 | (6.41, 13.73] | 750 | 24.96% |
|  | Q3 | (13.73, 25.7] | 751 | 24.99% |
|  | Q4 | (25.7, 288.14] | 751 | 24.99% |

**Appendix Table 4*.*** Adjusted association of flavonoids intake with periodontitis for sensitivity analysis.

| **Exposure** | **Unadjusted model** | **Adjust 1** | **Adjust 2** |
| --- | --- | --- | --- |
|  | Odds ratio (95% CI ) associated with periodontitis | | |
| **Total flavonoid intake** |  |  |  |
| Q1 | 1 (Ref) | 1 (Ref) | 1 (Ref) |
| Q2 | 0.63 (0.52, 0.77); <0.001 | 0.51(0.38, 0.67); <0.001 | 0.59(0.42, 0.83); 0.004 |
| Q3 | 0.57 (0.44, 0.73); <0.001 | 0.42(0.31, 0.58); <0.001 | 0.51(0.35, 0.73); 0.001 |
| Q4 | 0.71 (0.53, 0.94); 0.02 | 0.52(0.37, 0.73); 0.003 | 0.67(0.49, 0.91); 0.01 |

Unadjusted model: Non-adjusted model.

Adjust 1: Adjust for age, sex, race.

Adjust 2: Adjust for age, sex, race, body mass index, poverty income ratio, education levels, marital status, smoking status, alcohol consumption, hyperlipidemia, hypertension, diabetes mellitus and flossing.

**Abbreviations**: CI, confidence interval.

**Appendix Table 5*.*** Adjusted association of flavonoids intake with moderate/severe periodontitis.

| **Exposure** | **Unadjusted model** | **Adjust 1** | **Adjust 2** |
| --- | --- | --- | --- |
|  | Odds ratio (95% CI ) associated with periodontitis | | |
| **Total flavonoid intake** |  |  |  |
| Q1 | 1 (Ref) | 1 (Ref) | 1 (Ref) |
| Q2 | 0.71 (0.49,1.02); 0.06 | 0.57 (0.34, 0.95); **0.04** | 0.64 (0.40, 1.01); **0.05** |
| Q3 | 0.62 (0.43,0.90); 0.02 | 0.45 (0.28, 0.70); **0.005** | 0.55 (0.35, 0.84); **0.01** |
| Q4 | 0.85 (0.65,1.10); 0.20 | 0.59 (0.42, 0.83); **0.01** | 0.72 (0.53, 0.98); **0.04** |

Unadjusted model: Non-adjusted model.

Adjust 1: Adjust for age, sex, race.

Adjust 2: Adjust for age, sex, race, body mass index, poverty income ratio, education levels, marital status, smoking status, alcohol consumption, hyperlipidemia, hypertension, diabetes mellitus and flossing.

**Abbreviations**: CI, confidence interval.
