# Supplementary material for: Exploring the Nonlinear Relationship Between Dietary Flavonoid Intake and Periodontitis
Source: Int Dent J. 2024 Nov 14;75(2):716–26. doi: 10.1016/j.identj.2024.10.015 (PMC11976537; doi:10.1016/j.identj.2024.10.015)
Supplement: Supplementary file 3 [file mmc3.docx]

**Ethics approval**

The survey was performed by the National Center for Health Statistics (NCHS) and approved by the NCHS Institutional Review Board (IRB). All informed consents had been obtained from the eligible subjects before initiating data collection and NHANES health examinations.
